# Impact of Confined Water on the Electronic Structure of the SiO_2_ and WS_2_ Interface

**DOI:** 10.1021/acsami.4c19948

**Published:** 2025-02-13

**Authors:** Katherine L. Milton, Alexander Shluger

**Affiliations:** † Department of Physics and Astronomy and the London Centre for Nanotechnology, University College London, Gower Street, London WC1E 6BT, U.K.; ‡ WPI-Advanced Institute for Materials Research (WPI-AIMR), Tohoku University, 2-1-1 Katahira, Aoba-ku Sendai 980-8577, Japan

**Keywords:** band alignment, confined water, substrate interaction, 2D materials, density functional theory

## Abstract

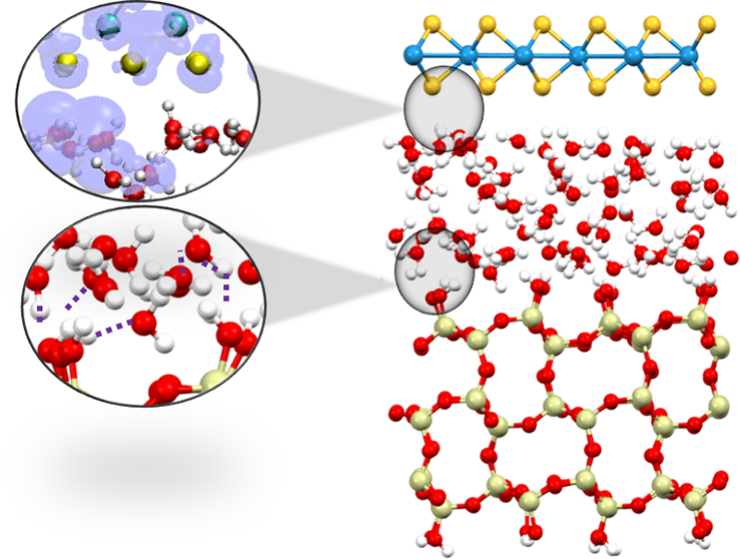

WS_2_/SiO_2_ heterostructures are commonly used in the production of field effect transistors, sensors and other devices. It is difficult to remove the water present on the SiO_2_ surface during the fabrication process. Using density functional theory simulations, we investigate how confinement between SiO_2_ and WS_2_ layers affects the water properties and how the presence of water affects the properties of WS_2_ film. Using *ab initio* molecular dynamics simulations we found that the confined water remains mobile but is structured by the interaction with WS_2_ and SiO_2_ with water protons drawn closer to both surfaces. The presence of 1–3 water layers does not significantly affect the band alignment between SiO_2_ and WS_2_ and the electronic properties of the WS_2_ monolayer. However, in-gap states caused by the dynamic rearrangement of water molecules could cause reduction of electron and hole mobility in the WS_2_ layers. This study provides insights into the impact of water on the electronic properties of WS_2_ under different environmental conditions.

## Introduction

In recent years, transition metal dichalcogenides (TMDs) have shown great potential in many applications, such as sensors, electrocatalysts in the hydrogen evolution reaction (HER), components in field effect transistors, and flexible electronic devices. In these applications, TMD films are deposited or grown on different substrates. However, how the interaction with a substrate affects the electronic properties of a TMD film is not well-known.

In this work we focus on WS_2_ which stands out as a stable two-dimensional semiconducting material with high electron mobility and an electronic band gap of ≈2.4 eV.^1^ In pursuit of using WS_2_ as a monolayer or a few-layer system in various applications, two primary approaches are used: direct growth on a substrate or exfoliation followed by deposition on the substrate. Exfoliation involves the separation of WS_2_ layers from the bulk material, and can be achieved by mechanical or chemical means. Chemical exfoliation offers versatile methods that use different supports and solutions, with water or water-based solutions commonly employed. The deposition of thin TMD films on substrates and/or further storage of heterostructures in ambient conditions can lead to the creation of thin water layers confined between a substrate and TMD films.^2^ This effect can be viewed both as detrimental to the performance of some devices and as a source of fascination for fundamental studies of nanoconfinement of liquids.^3,4^ In particular, understanding the behavior of water in close proximity to materials under different conditions is important for a broad range of applications and scientific research.

We aim to look at both sides of the coin: how confinement affects water and how the presence of water affects the properties of WS_2_ film. We investigate theoretically the structure and electronic properties of 1–3 layers of water confined between a WS_2_ monolayer and a hydroxylated crystalline SiO_2_ (silica) surface. This represents a broad case of strongly hydrophilic–weakly hydrophilic water confinement^3^ and is of interest for applications in transistor technology where WS_2_/SiO_2_/Si heterostructures produced by exfoliation and various growth methods have been extensively explored.^5−7^

Silica surfaces exposed to humidity are covered by Si–OH (silanol) groups, resulting in hydrophilicity of the surface and the presence of water at ambient temperatures and pressures.^8^ The hydrogen bonding of water to the silanol groups at the silica surface disrupts the hydrogen bonding network of liquid water. This causes the water to reorient and reduce mobility near the surface, although the exact structure of the water is still controversial.^9,10^ Experimental investigations have provided compelling evidence for the presence of confined water under ambient conditions on the surface of transition metal dichalcogenides (TMD) and silica.^11^ However, there are more experimental data on the properties of water confined on mica surfaces.^3^ In particular, there is evidence for charging and discharging of MoS_2_ and WS_2_ on mica by intercalating water films.^12^ Studies of MoS_2_ on sapphire have shown the ease at which water is intercalated between the materials resulting in changes in conductivity where water is present.^2^ The interface of MoS_2_ and SiO_2_ has had some insight from classical molecular dynamics (CMD) and experiment.^13^ However, the electronic properties of WS_2_ on silica substrates with water present remains unexplored.

In particular, one of the important characteristics that affects the applicability of TMDs in transistors is the band offset between the TMD valence and conduction bands and those of a gate dielectric. The band offset between amorphous silica and TMDs has been explored by internal photoemission (IPE) as a function of the TMD synthesis method (growth versus exfoliation).^14^ The observed variations in the band offset between MoS_2_ valence band maximum (VBM) and SiO_2_ conduction band minimum (CBM) have been attributed to the presence of water confined between the SiO_2_ and MoS_2_ as a result of deposition process. A combined X-ray photoelectron spectroscopy (XPS) and density functional theory (DFT) study has shown that the VBM and CBM fluctuate over time due to the movement of liquid water, highlighting the impact of the geometry of water on the electronic properties of the interface.^15^ However, IPE studies of WS_2_ on SiO_2_ as a function of the synthesis method^6^ found no significant change in band offset. The DFT and XPS studies have further confirmed a straddling band alignment between WS_2_ and SiO_2_.^16^ Other experiments using photoluminescence (PL) indicate that subtle interactions between water and TMDs may cause the existence of trap states.^17^

Despite these advances, it is still unclear how the water present at the TMD/H_2_O/SiO_2_ interface affects the electronic properties of each material and whether the electronic properties of the confined water depend on the number of water layers and their interaction with confining surfaces. From a practical perspective of the applications of TMDs in devices, important questions remain: (i) How the presence of water layers affects the adhesion of SiO_2_ substrate with WS_2_ film. (ii) Whether the presence of water affects the average band alignment between materials. (iii) Does the confined water remain mobile. (iv) Can fluctuations of water molecules affect electron mobility in WS_2_.

Here we investigate the properties of WS_2_/H_2_O/SiO_2_ heterostructures with different numbers of water layers using DFT and *ab initio* molecular dynamics (AIMD). This approach enables us to capture how the behavior of WS_2_ can change at different humidity conditions and to calculate the electronic structure and band offsets in the entire heterostructure. The results demonstrate that the interaction between the WS_2_, water and SiO_2_ layer, on average, results in a type I or the so-called straddling band alignment where the band gap of WS_2_ is completely contained within the much wider band gaps of SiO_2_ and water (see also ref (18)). Although perturbed, this band alignment is similar to the rigid band alignment. Thus, the presence of water does not significantly affect the band offset between SiO_2_ and WS_2_ and the electronic properties of the WS_2_ monolayer. However, the formation of localized, short-lived, in-gap states between H_2_O and WS_2_ may affect the electron and hole conductivity in WS_2_ layer. This study provides an atomistic insight into the structure and properties of confined water at the WS_2_/silica interface, contributing to our understanding of the impact of water on the electronic properties of WS_2_ under different environmental conditions.

## Methodology

### DFT Calculations

#### Properties of Individual Materials

Surfaces of amorphous silica films and silica glasses that can serve as substrates for the deposition or growth of TMDs under normal conditions are known to contain one silanol group per surface Si atom.^8^ It is commonly accepted that the (10 1̅) surface of the SiO_2_ crystalline phase α-cristobalite (α-C) represents a good mimic of the density and distribution of single silanol groups on an amorphous silica surface.^19^ The density of silanol groups on this surface (4.7 OH nm^–2^) is indeed similar to that of amorphous silica (4.5 OH nm^–2^), which is predominantly used in device manufacturing. We therefore use α-C as a model substrate in all further calculations. Based on previous simulations of the properties of silica surfaces, we expect that disordered locations of silanol groups characteristic of amorphous substrates will not affect our conclusions.^20,21^

We start by testing our methods on individual perfect materials. The 3 × 3 × 3 extension of the α-C bulk structure, the 6 × 6 cell of the WS_2_ monolayer and the 3 × 3 × 3 extension of the I_*h*_ bulk ice were calculated with DFT using the CP2K code.^22^ The double-ζ Gaussian basis set with the Goedecker-Teter-Hutter pseudopotential was used in all calculations. We used the PBE0-TC-LRC (Truncated Coulomb and Long Range Correction) exchange-correlation **(XC)** functional and the auxiliary density matrix method (ADMM) to speed up the calculations. The PBE0-TC-LRC functional uses a constant fraction of exact exchange in the short-range until a specified cutoff distance, Rc, at which it sets exact exchange to zero. In this work the truncation radius Rc = 2 Å and the fraction of Hartree–Fock (HF) exact exchange α = 25%. We note that the amount of HF exchange was not optimized to reproduce band gaps of the wide bandgap insulators H_2_O and SiO_2_ because the interface consists of three materials with different dielectric constants and different amounts of exchange will be needed to accurately describe each material. Therefore, optimizing the amount of exchange for one material at the expense of the others is not feasible. Standard PBE0-TC-LRC accurately describes the band gap of WS_2_ in both the pristine hexagonal and orthorhombic phases. The dispersion interaction was included using the D3(BJ) correction.^23^

The calculated geometric structures of the three perfect systems are in good agreement with experiment. The properties of the bulk α-C are discussed in more detail in ref (24) and the dependence of the properties of WS_2_ layers on the computational parameters is discussed in ref (25) The calculated Kohn–Sham and experimental band gaps and positions of the CBM relative to the vacuum level (V_0_) of perfect materials are summarized in Table 1.

**Table 1:** Experimental and Calculated Values of Band Gap and Conduction Band Minimum w.r.t Vacuum Level^a^

Reference	Material	Band Gap (eV)	V_0_ (eV)
Literature Values	α-Quartz SiO_2_^26^	9.65	–
	amorphous SiO_2_^27,28^	8.95	–0.75
	WS_2_^1^	2.4	–3.91–3.93
	Ice Ih^29^	9.4	–0.9
	Liquid Water^29^	9.0	–1.0
			
This Work	α-C SiO_2_ Slab	8.2	–0.6
	Liquid Water	7.65	–0.53
	hexagonal WS_2_	2.47	–2.34
	orthorhombic WS_2_	2.34	–3.89
	bulk α-C	8.57	–
	bulk Ice Ih	8.39	–

a The data for liquid water from this work shown in the table are obtained from 3 layers of water geometry optimized without any interface present.

#### Constructing the SiO_2_/H_2_O/WS_2_ Interface

The hydroxylated silica substrate was represented by a 3 × 2 × 1 α-C slab, with 12 silanol groups on either side of the slab. With the introduction of a vacuum gap on either side of the cell, its dimensions were 16.9 × 14.9 × 60 Å, with the SiO_2_ slab ≈10 Å thick.

To construct the SiO_2_/H_2_O interface, the water and silica surface were equilibrated separately using classical molecular dynamics (CMD) simulations, with the cell *x⃗* and *y⃗* dimensions fixed to the size of the silica cell. Then a 3D periodic cell was constructed by attaching the water boxes on either side of the silica slab with the length of the cell along *z⃗* chosen so that, far from the surface, the average water density was 1.00 gcm^–3^. We used the standard 12/6 Lennard-Jones potentials with the parameters given by the INTERFACE force field^19^ for the Si, O, and H atoms in silica and the TIP4*P*/2005f^30^ force field for H and O atoms in water. Geometric mixing rules were applied to describe interactions between water and silica. Using the time step of 0.5 fs, and T = 298.5 K CMD of this system was run for 10 ns in an NVT and then in an NVE ensemble, followed by another 10 ns CMD run in an NVT ensemble. This allowed us to achieve accurate bulk water densities in the system and reproduce the characteristic layered water structure near the interface.^9^

This system corresponds to the interface between a thick water layer and a silica surface. However, in deposition experiments, exfoliated TMD layers are deposited on a silica surface exposed to ambient conditions. Based on the data reviewed in ref (8) we assume that, depending on humidity and anneal temperature, the silica surface may have between 1 and 3 adsorbed water layers under such conditions. Based on this insight, we have constructed three SiO_2_/H_2_O cells with 1, 2, and 3 water layers, respectively. The initial structures of these cells were produced by truncating the equilibrated cell at the troughs of the radial distribution function (RDF) of the water layer as a function of distance from the surface (see Figure S1). To equilibrate this system, a 100 Å vacuum gap was added to the truncated SiO_2_/H_2_O systems with 1–3 water layers and the same CMD procedure was repeated again, where these systems were equilibrated with an NVT and NVE ensemble for 5 and 10 ns, respectively, at 298.5 K. The adsorbed water layers remain intact on the surface, confirming the reliability of the force fields used. A detailed description of the structure and dynamic characteristics of this system is provided in ref.^31^

To construct the SiO_2_/H_2_O/WS2_2_ interface, the bottom layer of water was removed, so the layers of water are only on one side of the SiO_2_ slab, and the bottom silica layer was left hydroxylated. This was done to ensure that the resulting 2D slabs with 1, 2, and 3 water layers are amenable for AIMD simulations of the total system.

To accommodate the WS_2_ monolayer in these cells, the original WS_2_ unit cell was transformed into an orthorhombic cell to ensure a good fit and minimal strain when added to the CMD-optimized SiO_2_/H_2_O system. The geometry of the 5 × 3 orthorhombic WS_2_ structure was optimized using the PBE0-TC-LRC XC functional and the resulting band gap is given in Table 1. The electronic structure of TMDs is known to depend on strain. However, introducing strain is inevitable when creating interfaces because of a lattice mismatch between the materials of the heterostructure. We found that the minimum strain between SiO_2_ and WS_2_ with fewer than 700 atoms in a periodic cell can be achieved when a 5 × 3 orthorhombic unit cell of WS_2_ was rotated 90° with respect to the 2 × 3 × 1 SiO_2_ slab. This leads to 2.62% strain (compression) in the **a** direction and −5.69% strain (tension) in the **b** direction of WS_2_. A further discussion on the impact of strain is in the Supporting Information (Figure S2).

The strained, orthorhombic WS_2_ monolayer was then added to the SiO_2_/H_2_O interface and a vacuum of at least 35 Å was introduced. The geometry of the whole system was optimized using DFT and a PBE-D3(BJ) functional at different WS_2_ slab distances from the water surface of H_2_O/SiO_2_ to increase the efficiency of the calculation.

**Figure 1:**
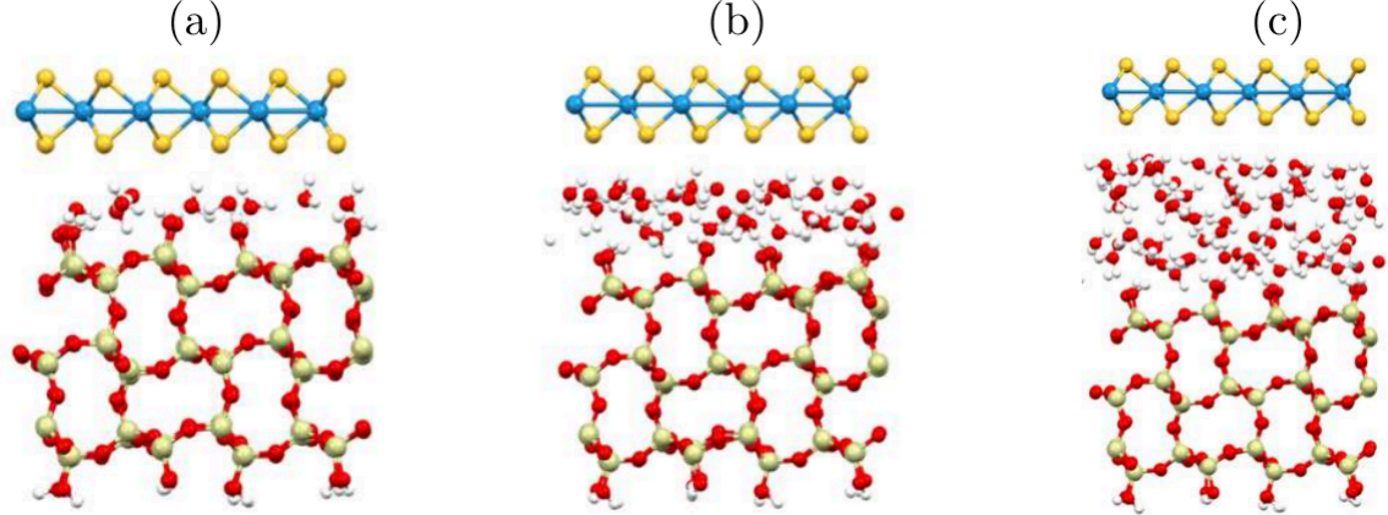
Snapshot geometric structures of the SiO_2_/H_2_O/WS_2_ interfaces with (a) 1 layer of water, (b) 2 layers of water, and (c) 3 layers of water. colors: blue = W, yellow = S, red = O, white = H, beige = Si.

The optimized structure was then used as the starting geometry for the AIMD calculations in an NVT ensemble maintained at 400 K with a Nose-Hover thermostat. In these simulations, we used the PBE-D3 functional because it provides a good geometry for WS_2_ and SiO_2_ although it is known to overbind water.^32−34^ The high temperature was chosen because increasing the temperature to 350–400 K was shown to overcome this issue.^35,36^ The equilibration time for AIMD was 4 ps, after this time, the system’s total energy remained constant. This was followed by the 12.5 ps production runs and snapshots were taken every 0.5 ps. Examples of snapshot geometries for the three structures are shown in Figure 1.

Single-point energy calculations using the PBE0-TC-LRC + D3 XC functional and a dipole correction were performed for the 26 snapshots. The geometric structures captured in these snapshots were not reoptimized with PBE0-TC-LRC to keep the non-0K configurations of water, but the hybrid functional provided a better description of the band gap and, consequently, the band alignment.

To better understand and provide comparison to how WS_2_ interacts with water without the presence of SiO_2_, WS_2_/H_2_O systems were created by removing the SiO_2_ slab from the SiO_2_/H_2_O/WS_2_ interfaces. AIMD was then run using the same methodology as described above. This involved a 4 ps initial run which allowed the water to restructure from the previous configuration, and then a 12.5 ps production run with snapshots taken every 0.5 ps with a single-point calculation PBE0-TC-LRC + D3. We note that the water did not evaporate at this interface at 400 K.

#### Snapshot Analysis

In total, 26 snapshots of the AIMD trajectory were analyzed and averaged. To calculate the band alignment of the system, a reference value is required. Previous work investigating the band alignment of water and semiconductors have used averaged electrostatic profiles referenced to the vacuum level with average bulk potentials used to align conduction bands to determine band alignment.^37^ However, in our work, the small amount of water used means that the bulk water potential could not be easily determined. Therefore, the local density of states (LDoS) was used to determine the band alignment.

The alignment of the bands in the SiO_2_/H_2_O/WS_2_ heterostructure was determined by averaging the LDoS with the vacuum level as a reference. The average LDoS was spatially resolved along the *z⃗* axis, perpendicular to the interface. The energy resolution of the states was 0.1 eV, with a 0.5 Å smearing applied to the spatial resolution using a Gaussian and previously reported methodology.^38^

The dynamic nature of the interface introduces variability in the electronic states across different time snapshots, complicating the determination of the local band gap. As a result, the density of states associated with dynamic eigenstates is reduced due to the temporal averaging. The local VBM and CBM were identified by analyzing the density of states within the averaged LDoS. A threshold-based approach was employed to define the local VBM and CBM, where the states were considered only if their LDoS contribution exceeded a predefined cutoff value, corresponding to a specific state density.

This threshold was used to modulate the sensitivity to detect the local CBM and VBM, allowing the capture of subtle variations in the alignment of the band that may arise from the dynamic interaction between water molecules and the WS_2_ surface. The higher threshold of 0.5 provided average values, revealing larger band gaps for SiO_2_ and H_2_O, while the lower threshold of 0.1 offered greater sensitivity to spatially dependent interactions between WS_2_ and H_2_O. The impact of these threshold values and their implications for band alignment will be discussed in detail in the Results and Discussion section.

The inverse participation ratio (IPR) was calculated from the snapshots to quantify the degree of localization of the eigenstates. The IPR methodology, previously detailed in the literature^39^ uses atom-centered base sets in CP2K to assess the localization of eigenstates. The IPR values range from 1, representing highly localized states, to 0, indicating completely delocalized states.

The adhesion energy was calculated as
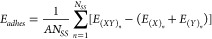
1where *A* is the surface area, *E*_*XY*_ is the energy of the entire interface, *E*_*X*_ and *E*_*Y*_ are the total energies of the individual materials at the interface separately calculated from the same snapshot geometry, *n* is the number of a snapshot and *N*_*SS*_ is the total number of snapshots. In some literature, a factor of  is added to account for the two surfaces at an interface. However, since most papers do not include this term, we have excluded it for easier comparison.

Average adhesion energy calculations for interfaces involving water use snapshots from an AIMD trajectory. In contrast, for the WS_2_/SiO_2_ interface, a single optimized geometry is used; therefore, this adhesion energy is not averaged.

## Results and Discussion

### Interface Structure and Adhesion Energy

We start by calculating the binary interfaces to compare them with the previous literature. The low adhesion energy between WS_2_ and several water layers seen in Table 2 shows that water is physisorbed on WS_2_, and that adhesion increases with the number of water layers. This mild physisorption is expected as experimental evidence shows that WS_2_ is mildly hydrophilic.^40^

**Table 2:** Average Adhesion Energy of WS_2_ interface, and Average Adsorption Energy with Respect to the Number of Water Layers Present at the Interface

Interface	Water Layers	Adhesion Energy (Jm^–2^)
WS_2_/H_2_O	1	–0.0531 (±0.005)
	2	–0.146 (±0.008)
	3	–0.164 (±0.010)
		
WS_2_/H_2_O/SiO_2_	1	–0.188 (±0.011)
	2	–0.196 (±0.005)
	3	–0.194 (±0.007)
		
WS_2_/SiO_2_	0	–0.250

**Figure 2:**
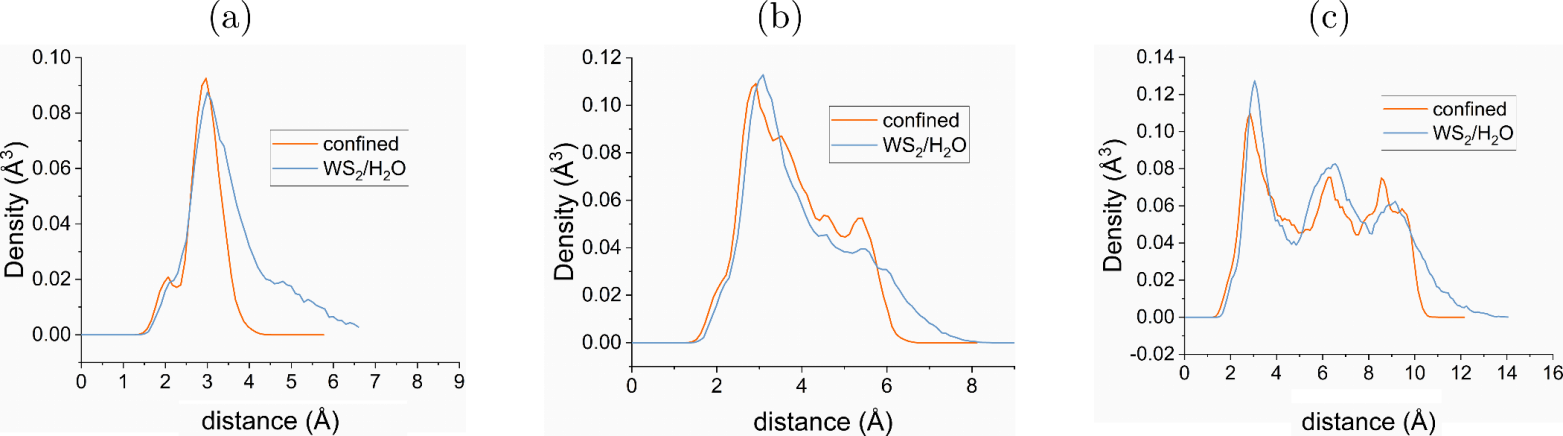
Averaged 1D density profile of water over AIMD frames, the 0 point is the sulfur layer closest to water of WS_2_. Confined water at the SiO_2_/H_2_O/WS_2_ interface is in orange, and water from WS_2_/H_2_O interface in blue. (a) 1 layer of water, (b) 2 layers of water, and (c) 3 layers of water.

Before discussing the adhesion of WS_2_ and water inside the SiO_2_/H_2_O/WS_2_ system, we comment on the structure of confined water layers. A detailed analysis of this structure and the role of different interactions is given in ref.^41^ Briefly, the water remains mobile but is structured by both WS_2_ and SiO_2_ with water protons, H_*w*_, drawn closer to both surfaces. The interactions with silanol groups significantly affect the structure of the water in a monolayer (Figure 2). In the case of 2 and 3 layers, the water near the interfaces also appears to be highly structured. The interaction of water with WS_2_ competes with the silanol groups and induces some reorientation of the water molecules, with H_*w*_ turning toward WS_2_ in confinement. We note that, although the water confinement between WS_2_ and SiO_2_ leads to increased structuring, most of the structure is still determined by the water interaction with the silanol groups. The shoulders seen at about 2.2 Å from the sulfur plane of the WS_2_ surface correspond to the H_*w*_ orientation toward that surface and give an estimate of the distance between the WS_2_ surface and water layer.

The interaction of water with the other two materials can be characterized using the adhesion energy. For the SiO_2_/H_2_O/WS_2_ interface, similar to the WS_2_/H_2_O interfaces, adhesion energy increases with the number of water layers; however, the confined system exhibits a significantly higher adhesion strength. Similar trends have been reported for graphene/H_2_O/SiO_2_ systems, though the adhesion energy for graphene is slightly lower, as graphene is more hydrophobic than WS_2_.^42,43^ It is observed that the adhesion energy stabilizes at three water layers at the SiO_2_/H_2_O/WS_2_ interface. This indicates that beyond a certain distance, additional water molecules contribute negligibly to the energy gain due to the limited interaction with the WS_2_ surface. This suggests that the interaction between WS_2_ and H_2_O is short-range, being dominated by van der Waals (vdW) forces.

Atomic force microscopy (AFM) measurements performed on the MoS_2_/SiO_*x*_ system provide a relevant comparison.^44^ In these experiments, at a temperature of −15 °C, where it was expected that water would be confined at the interface, the adhesion energy was measured at 0.152 Jm^–2^, which is comparable to the adhesion energies reported in Table 2. The rise in temperature and, thus, the reduction in the amount of interfacial water resulted in an increase in the adhesion energy. This agrees with the increase in adhesion energy when water is removed leaving only the hydroxylated SiO_2_ slab and WS_2_ in contact also observed in our work (see Table 2).

### Rigid Band Alignment

To understand the electronic structure of the interface, we first calibrate our results to known values and create a rigid band alignment (RBA) diagram. This popular method is based on the bulk characteristics of individual systems. We can then compare the results with a more realistic system where interactions at interfaces may change this alignment.

In our study, the vacuum level serves as the common reference point for the RBA across all materials. To align the materials to this reference, two key values are required for each material: the CBM relative to the vacuum level (V_0_), which corresponds to the electron affinity, and the band gap. V_0_ ensures that all materials are aligned consistently with respect to the vacuum level, thereby providing a uniform reference for comparing electronic properties across different materials.

**Figure 3:**
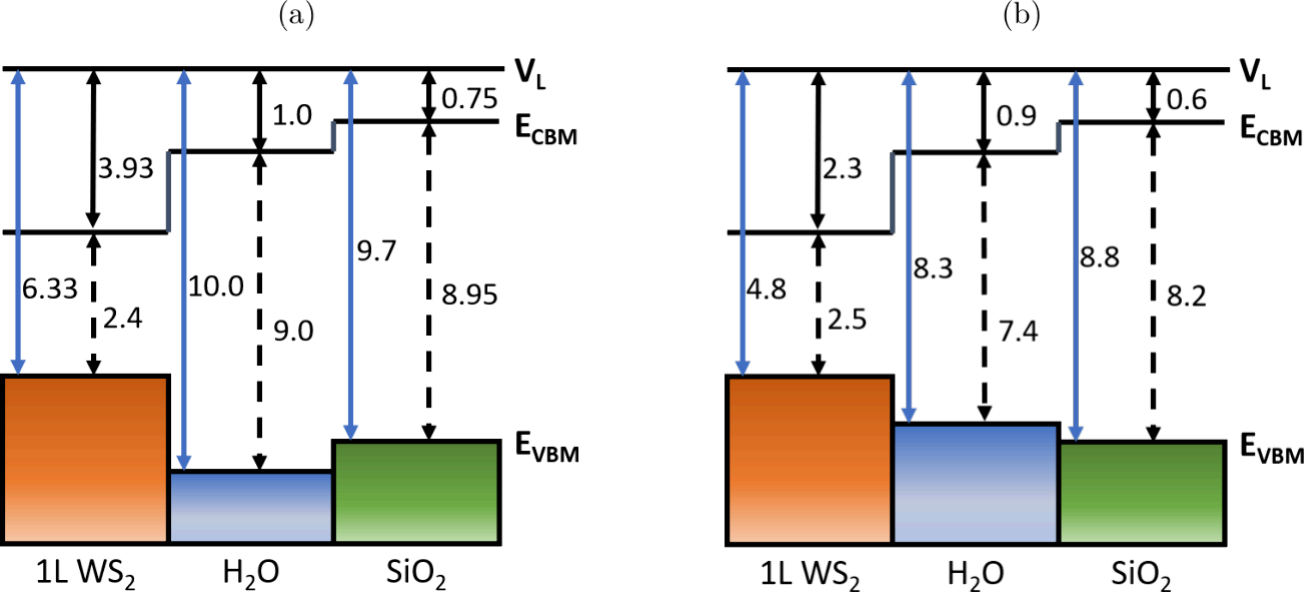
Rigid band alignment of the WS_2_/H_2_O/SiO_2_ interface with reference to the vacuum level (V_*L*_), shown for (a) experimental values from the literature and (b) values calculated in this work using the PBE0-TC-LRC functional (see Table 1 for detailed comparison and references).

The experimental values for the RBA are collated in Table 1 and depicted in Figure 3a. Unlike the α-C phase used in this study, the amorphous and alpha-quartz phases of SiO_2_ have been well studied and, therefore, have more accurate band gap values in the literature. The bulk band gaps calculated in our work are compared with the literature values for α-C and ice I_*h*_ to understand how the chosen XC functional affects electronic properties. The previously calculated α-C is close to the experimental value of amorphous SiO_2_, with only a slight underestimation; see Table 1. For ice I_*h*_, the band gap is underestimated by 1.01 eV. As discussed in the methodology section, this is due to the 25% HF exchange used in this work.

The parameters for the RBA based on the data calculated in this work, seen in Figure 3b, are obtained using slab models where the band gap is reduced with respect to the bulk value. To understand how the SiO_2_ band gap can change due to the small slab thickness used in the simulations, we refer to the work on SiO_2_ films, where the experimental band gap was determined to be 6.7 eV,^45^ which is much smaller than our slab value. We ascribe this to the difference in the film structure and to the presence of the interface with Ru in the experimental measurements.

The choice of XC functional and the amount of water lead to underestimating the water band gap too, which changes the offset between water and silica compared to the bulk experimental values; see Figure 3b. However, the description of water and silica as dielectrics and WS_2_ with a straddling band alignment is consistent. The calculated V_0_ values match fairly well with the values in the literature, so we expect the CBM alignment to be fairly accurate.

### Average Band Offset

The band alignment in the system of interacting layers was examined using the averaged LDoS projected on atoms along the axis perpendicular to the interface slabs. The local VBM and CBM were identified by analyzing the eigenvalues below and above the Fermi level, respectively. For a state to be considered the VBM or CBM at a particular position within the stack, the averaged density of states of the atoms contributing to that state must exceed a specified threshold. This approach enables the LDoS to be adjusted, either to resolve finer features or to increase the threshold for a clearer LDoS representation.

**Figure 4:**
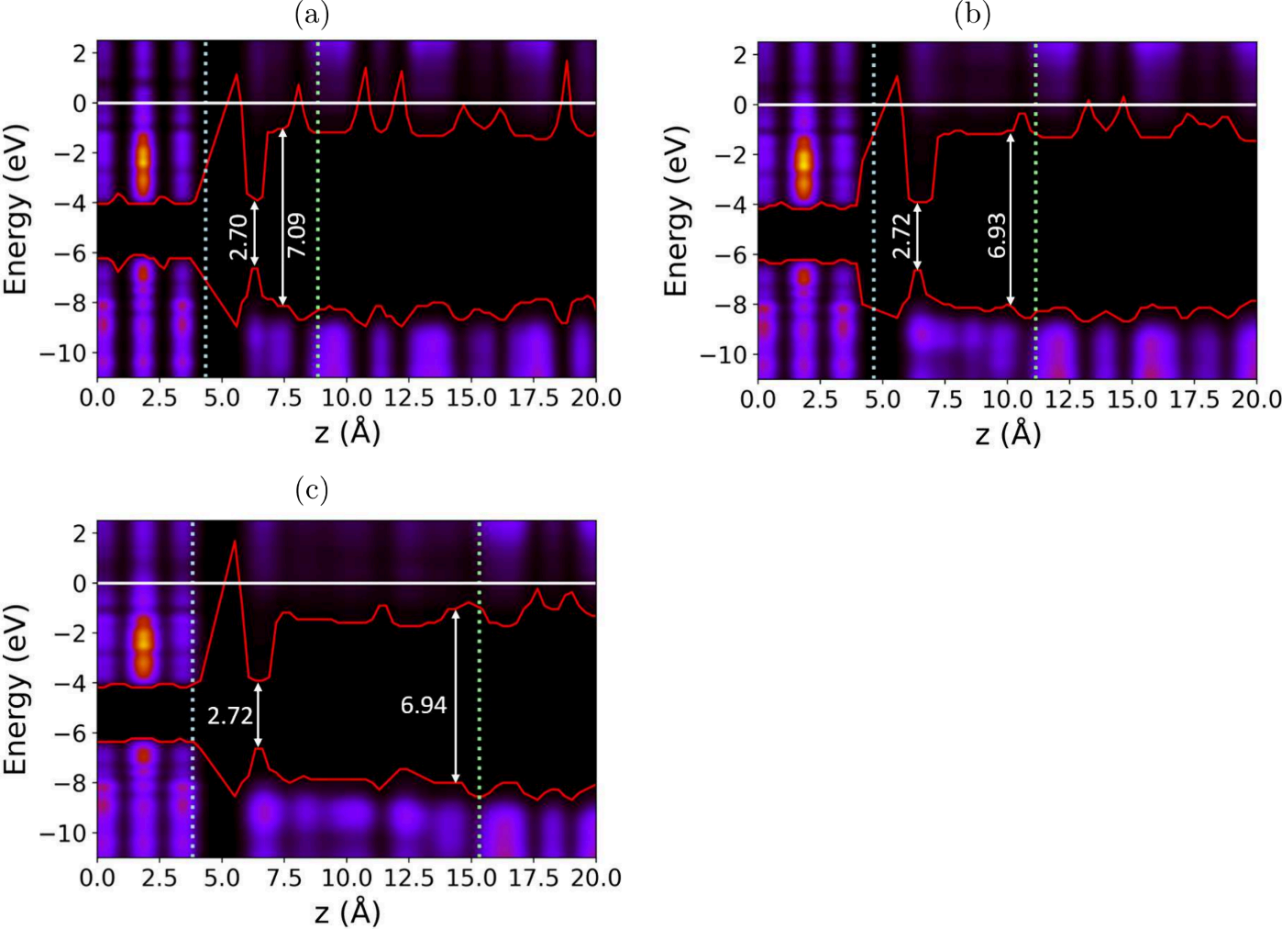
Band alignment derived from the local density of states (LDoS) at the SiO_2_/H_2_O/WS_2_ interface, applying a threshold cutoff of 0.5. Red lines indicate the VBM and CBM at the interface. The color map represents the LDoS in arbitrary units, illustrating the atomic contributions to the eigenstates across the energy spectrum. The blue dotted line marks the termination of WS_2_, while the green dashed line indicates the start of the SiO_2_ slab. Arrows denote the band gaps of water in contact with WS_2_ and SiO_2_, with corresponding values in eV. The white line at 0 eV corresponds to the vacuum level. (a) 1 layer of water, (b) 2 layers of water, (c) 3 layers of water.

In Figure 4, a threshold of 0.5 was applied, representing a relatively high cutoff. Here, significant variations in the local VBM and CBM are observed, particularly within the SiO_2_ slab. Due to the crystalline nature of SiO_2_, the atomic positions exhibit minimal average displacement, resulting in a noticeable “layering” along the *z*-direction: when there are no atoms contributing substantially to the LDoS, the electron density falls below the threshold. Further notable drops occur at the WS_2_/H_2_O interface, where water does not form bonds with WS_2_, leading to a small gap at the structure. A similar effect is observed at the SiO_2_/H_2_O interface, although the VBM and CBM drop is less pronounced, as water is drawn closer to the SiO_2_ surface through hydrogen bonding with silanol groups. A clearer figure showing the alignment of the position of the averaged atoms with respect to LDoS can be seen in SI Figure S4.

In contrast to RBA, the introduction of interfaces between SiO_2_ and water leads to a reduction in their respective band gaps, which decrease to 7.25–7.5 eV. This suggests that the electronic properties of the system are altered by the presence of these interfaces, as demonstrated in Figure 4a-4c. Although the band gap of WS_2_ also decreases by 0.16 eV, the variation in its band gap is minimal when water is introduced, which aligns well with experimental findings on WS_2_ band alignment.^6,15^ Previous studies have reported differences in the band gap and band offsets in MoS_2_/WS_2_ systems in contact with water. These variations have been attributed to factors such as structural perturbations, dipole changes, and electron density overlap, which may collectively explain the observed reduction in the band gap.^46−48^

For all layers of water, the offset of the WS_2_ and SiO_2_ bands and the WS_2_ band gap remain consistent, with a valence band offset (VBO) of 1.91–2.18 eV and a conduction band offset (CBO) of 3.14 eV. To understand the offset results calculated in this work, we compare them with the experimental values, as seen in Table 3. The experimental literature shows a consistent straddling band alignment. Still, the offset varies depending on the experimental technique used, with IPE showing much larger VBO and smaller CBO compared to XPS. Our results are more consistent with the XPS values. However, the VBO is consistently underestimated due to the reduced band gap in SiO_2_, as previously discussed.

**Table 3:** Average Electronic Properties of the WS_2_/H_2_O/SiO_2_ System in eV^a^

System	Band Gap	Average CBO	Average VBO
rigid	2.34	2.42	2.86
1 layer^b^	2.18	3.14	2.18
2 layers	2.18	3.14	1.91
3 layers	2.18	3.14	1.91
HSE06^16^	2.24	2.58	3.17
XPS and STS^16,51^	2.38	2.70	3.97
IPE^c^^6,7^	n/a	≈1.4	5.1
PEEM^d^^52^	n/a	3.3	4.0

a The CBO and VBO are shown for the calculated and measured in the literature values between SiO_2_ and WS_2_.

b From LDoS calculations using 0.5 threshold.

c IPE used a 8.9 eV SiO_2_ bandgap, we have applied a 2.4 eV WS_2_ bandgap.

d PEEM used a 9.7 eV SiO_2_ band gap.

The most accurate measure of band alignment should be coming from IPE, where the alignment of the WS_2_ VBM to the SiO_2_ CBM is measured directly when the two materials form an interface. This alignment has been experimentally determined to be 3.8 eV.^7^ In contrast, our calculations yield a value of 5.32 eV. One factor influencing the band alignment of WS_2_/SiO_2_ may be the conditions under which WS_2_ was measured, such as the presence of dopants which will impact the work function of WS_2_, the formation of adsorbate layers on the WS_2_ surface, or the charging of WS_2_ layers under photon excitation.^49,50^ We also note that the ionization energy of WS_2_, obtained from the averaged projected density of states (PDoS) of our system (SI Figure S3), is approximately 6 eV, which falls within both experimental and theoretical ranges. Furthermore, we note that the LDoS V_0_ values for all water layers in Figure 4 are ≤1 eV, consistent with the experimental values, indicating that the CBO in this work is accurately described. Finally, the WS_2_ V_0_ for IPE is 2.3 eV (with SiO_2_ V_0_ equal to 0.9 eV), smaller than literature values in Table 4, further highlighting experimental conditions as a significant factor.

**Table 4:** Literature Values of Ionization Potential (IP), Work Function (WF), and V_0_ of WS_2_ in eV

Method	Ionization Energy	Work Function	V_0_	Synthesis/Method	Substrate
IPE^a^^7^	4.55–4.9	–	–	MBE/MOCVD	SiO_2_/Si
UPS^53^	–	4.95	–	hydrothermal, powder	water (nanoflower)
I_*D*_-V_*G*_^b^^54^	6.9	4.6	4.5	exfoliation	SiO_2_/p+ Si
PEEM^55^	5.74	–	–	CVD	SiO_2_/p+ Si
KPFM^1^	5.48	4.75–4.13	3.91–3.93	CVD, exfoliation	Au and Al
G_0_W_0_^56^	6.09	4.85	3.6	SOC	–

a The IPE technique values have been extrapolated using SiO_2_ V_0_ to be between 0.75–0.9 eV.

b The I_*D*_-V_*G*_ ionization potential is extrapolated with WS_2_ band gap equal to 2.4 eV.

However, we must also consider that the difference of 1.52 eV between our results and IPE may arise from our choice of XC functional, since our calculated SiO_2_ band gap is underestimated by 1.67 eV compared to the value used in the IPE study. The discrepancy in band gap can also be highlighted by comparing the PEEM and IPE studies in Table 3, where varying band gap assumptions impact band-offset outcomes. Here, the CBO and VBO influenced by the assumption of an 8.9 eV band gap for SiO_2_ in IPE versus a 9.7 eV band gap in PEEM.

### Water Band Gap

The structure of the nanoconfined water layer is determined by the interaction with the confining surfaces. We investigated how this confinement affects the water’s band gap. Understanding water’s properties is particularly challenging because of the influence of nuclear quantum effects (NQE), which still remains controversial. We used the results of Bischoff et al.^29^ as a reference.

The band gap of the confined water at the SiO_2_/H_2_O/WS_2_ interface was determined using LDoS, as shown in Figure 4. In particular, local variations in the VBM and CBM are influenced by the proximity of the surfaces of WS_2_ and SiO_2_. For all water layers, the band gap of H_2_O decreases near the WS_2_ surface but increases as it approaches the SiO_2_ surface. At the SiO_2_ interface, the band gap is similar to that of bulk I_*h*_ phase. This increase of the band gap results in minimal band offset between H_2_O and SiO_2_, caused by the strong hydrogen bonding between H_2_O and the SiO_2_ surface, which induces structuring of the water molecules near the interface. The reduction in the band gap near the WS_2_ surface is hypothesized to arise from the formation of in-gap states between H_2_O and WS_2_ discussed in detail below.

To assess the effects of confining surfaces on the water electronic structure, we analyzed changes in the water band gap upon removal of the interface. This was done using geometric structures of water extracted from interface snapshots, calculating the band gap for each configuration, and averaging the results. For a single layer of water the average band gap (8.12 eV) remains relatively unchanged compared to the confined system. However, for two (5.19 eV) and three (5.34 eV) layers of water, a significant reduction in the band gap is observed when the interfaces are removed. The LDoS in Figure S5 shows that, depending on the threshold value used, there is some spatial dependence of the band gap close to where the SiO_2_ surface was. This indicates that the change in band gap is caused by the impact of broken hydrogen bonding at the interface. The rearrangement of water molecules by surface silanol groups leads to a change in the dipole moment, which in turn affects the system’s overall dipole when the interface is removed.

Overall, the pronounced changes in the water band gap under confinement, compared to those in the unconfined case, underscore the strong interactions between water and the confining materials, specifically the hydrogen bonding between water and silanol groups.

### Work Function Shift

It is also interesting to see how the presence of water and SiO_2_ layers affect the work function of WS_2_. Previous studies have shown that thermalized water can alter the work function of metals over time, depending on the orientation of the water molecules and the resulting dipole interactions with the metal surface.^57,58^ The work function changes at interfaces are linked to factors such as reduced electron spillover into the vacuum and surface roughness.^59^ The shift in work function can be significant, with the literature reporting changes (*Δϕ*) of up to 1.4 eV in systems such as MgO/Ag.^60,61^

We find that the confined water interface shifts the WS_2_ work function (*Δϕ*) on average by −0.10 (±0.15), −0.20 (±0.20), and −0.26 (±0.23) eV for 1, 2, and 3 layers of water, respectively, which is notably smaller compared to typical values for metal oxide/metal or metal/water interfaces.

To assess the impact of water confinement, we compared the *Δϕ* values at the WS_2_/H_2_O/SiO_2_ interface with those for the WS_2_/H_2_O system. The WS_2_/H_2_O interface was calculated using the same methodology, with an AIMD calculation and 25 snapshots analyzed to get an average. The calculated *Δϕ* values for 1, 2, and 3 water layers in the WS_2_/H_2_O system are −0.10 (±0.14), +0.021 (±0.14), and −0.097 (±0.21) eV, respectively. This comparison shows that for two and three water layers, the magnitude of *Δϕ* is significantly reduced in the WS_2_/H_2_O system compared to the confined WS_2_/H_2_O/SiO_2_ interface. However, for a monolayer of water, the *Δϕ* values are comparable between both interfaces. These results suggest that, while the interaction between WS_2_ and H_2_O is generally weak, the introduction of confinement notably enhances this interaction. Additionally, we note that the work function values of WS_2_ are likely to vary with relative humidity, which should be considered in experimental measurements.

### Effects of Water Fluctuations

Our AIMD results show that water remains mobile in the confined state and exhibits reorientations. The ensuing dipole fluctuations can affect the electronic structure of the heterostructure by introducing some short-lived states, as discussed for water/metal interfaces.^58^ The adsorption of water has been shown to significantly affect the electron and hole conduction of WS_2_ nanotubes^62^ and it has been suggested that adsorbed water can act as trapping and scattering centers for free carriers.

**Figure 5:**
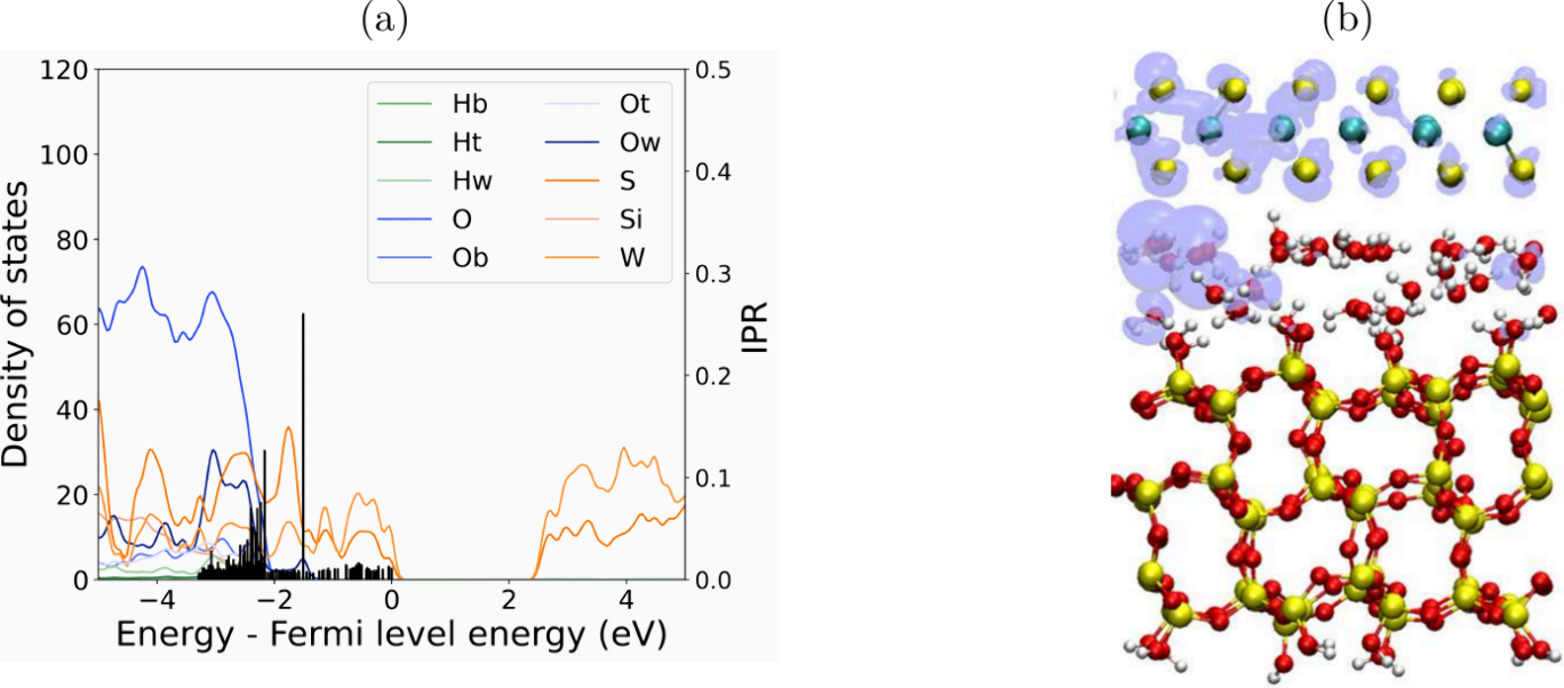
(a) PDoS for one of the AIMD snapshots for the SiO_2_/H_2_O/WS_2_ interface with two layers of water showing the IPR analysis of the valence band states. (b) The molecular orbital of the in-gap state with the highest IPR value, isosurface: 0.01.

The interface states of confined water at the H_2_O and WS_2_ interface were analyzed by examining the time evolution of the PDoS from simulation snapshots. The degree of localization of in gap states was analyzed by calculating the inverse participation ratio spectrum. Several of the states have high IPR values, indicating greater electron localization, as seen in Figure 5a for one of the snapshots. Similar states form in many snapshots, but the energies of the localized states vary significantly, as can be seen in SI Figure S6. The molecular orbital corresponding to the peak with the highest IPR is illustrated in Figure 5b. This and the PDoS analysis revealed the creation of electronic states with significant contributions from both water molecules and the WS_2_ valence band states in PDoS. These fluctuations highlight the effect of the water configurations and consequent dipole changes on the interfacial electronic properties. Across all water layers, the formation of short-lived in-gap states was observed in the region between the VBM of WS_2_ and that of H_2_O. These transient in-gap states became more frequent with increasing water layer thickness and are typically lasting less than 500 fs.

**Figure 6:**
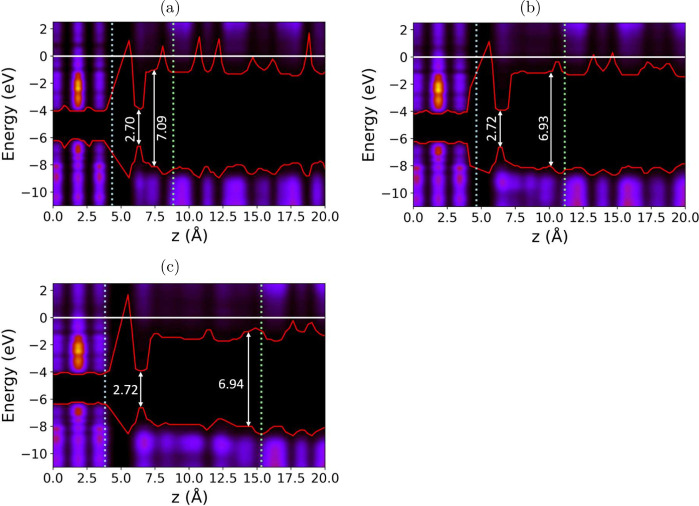
Band alignment with a 0.1 cutoff threshold at the SiO_2_/H_2_O/WS_2_ interface, red lines show the VBM and CBM of the interface, the surface on the right indicates the contribution of the atoms to the eigenstate at each energy. The diagram on the left is a scaled diagram of the average interface position. (a) 1 layer of water, (b) 2 layers of water, (c) 3 layers of water.

To investigate the effect of these dynamic processes on band offsets, we reduced the LDoS cutoff threshold to 0.1, thus increasing the sensitivity of band alignment, as shown in Figure 6. The resulting band alignment at the H_2_O and WS_2_ interface reveals a minimal offset near the WS_2_ surface across all water layers. This reduced band offset is spatially confined, occurring primarily in the water interfacing with the WS_2_ surface, while water molecules farther away from WS_2_ exhibit a wider band gap. Furthermore, there is no observed offset between SiO_2_ and the water adjacent to it.

To further examine the interaction at the interface, we analyzed the charge transfer between WS_2_ and H_2_O at the WS_2_/H_2_O/SiO_2_ interface. This charge transfer exhibits a similar spatial dependence on the water layers, as shown in SI Figure S7. Although the overall magnitude of charge transfer remains relatively small due to spatial averaging, these results indicate an interfacial interaction between water and WS_2_.

We note that electron and hole scattering on these dynamic localized states could be a mechanism responsible for the reduction of conductivity in WS_2_ nanotubes observed in ref.^62^ Similar scattering mechanisms in 2D transistors have recently been considered in ref^63^ The difference in electron transport properties of single-layer WS_2_ on SiO_2_ measured in air and vacuum has been attributed to the presence of water and oxygen gas, but is difficult to disentangle into components.^64^

## Conclusions

This work offers an insight into the impact of confined water within the WS_2_/H_2_O/SiO_2_ heterostructure, exploring different numbers of water layers using DFT and *ab initio* molecular dynamics. The results demonstrate that the confined water remains mobile but is structured by both WS_2_ and SiO_2_ with water protons drawn closer to both surfaces. The adhesion between WS_2_ and water layers is dominated by weak hydrophilic interactions. The structuring of water does not induce significant shifts in the band alignment at the interface compared to the rigid band alignment. This is confirmed by electronic structure calculations that demonstrate that the interaction between WS_2_, water, and SiO_2_, on average, results in a type I or the so-called straddling band alignment, in agreement with previous IPE measurements.^14^ The band gap of WS_2_ is completely contained within the much wider band gaps of SiO_2_ and water. The presence of water does not also significantly affect the average band offset between SiO_2_ and WS_2_ and the electronic properties of the WS_2_ monolayer. The work function of WS_2_ shifts by 0.1–0.26 eV with the introduction of confined water with the extent of the shift depending on the number of water layers. Localized short-lived in-gap states identified at the confined water interface between H_2_O/WS_2_ surfaces may affect the electron and hole conductivity in WS_2_ layers. To understand the importance of this effect, carrier mobility measurements in WS_2_ at water freezing temperatures and above room temperature will be useful. More studies are needed to understand the impact of confined water under experimental conditions in realistic WS_2_ systems that include interface defects.

## Supporting Information Available

Radial distribution function, strain of WS_2_, Average PDoS, water LDoS, further IPR analysis, charge transfer (PDF)
