# Supplementary material for: Impact of Confined Water on the Electronic Structure of the SiO2 and WS2 Interface
Source: ACS Appl Mater Interfaces. 2025 Feb 13;17(8):12841–51. doi: 10.1021/acsami.4c19948 (PMC11873964; doi:10.1021/acsami.4c19948)
Supplement: Supplementary file 1 — am4c19948_si_001.pdf [file am4c19948_si_001.pdf]

Supporting Information: Impact of confined water on the electronic structure of the SiO_2 and WS_2 interface

Katherine L. Milton[*,†] and Alexander Shluger[*,†,‡]

†*Department of Physics and Astronomy and the London Centre for Nanotechnology, University College London, Gower Street, London WC1E 6BT, UK*

‡*WPI-Advanced Institute for Materials Research (WPI-AIMR), Tohoku University, 2–1-1 Katahira, Aoba-ku, Sendai 980-8577, Japan*

E-mail: katherine.milton.20@ucl.ac.uk; a.shluger@ucl.ac.uk

Supporting Information

Radial Distribution Function

The radial distribution function (RDF) was calculated from the CMD run of $H_2O/SiO_2/H_2O$ between silanol H (H_t) and oxygen water (O_w), as seen in Figure S1. The height and narrowness of the initial peak, highlighted in red, show that the water close to the silica surface is structured and tightly bound to the surface, indicating a monolayer of water. We then see a shorted and broader second peak, highlighted in blue, indicating that the water is still structured in the second layer. The third peak, highlighted in orange, does not show a strong peak but resembles the more flat distribution of bulk water; therefore, this was chosen as the closest approximation to bulk water without unnecessarily extending the water for AIMD calculations.

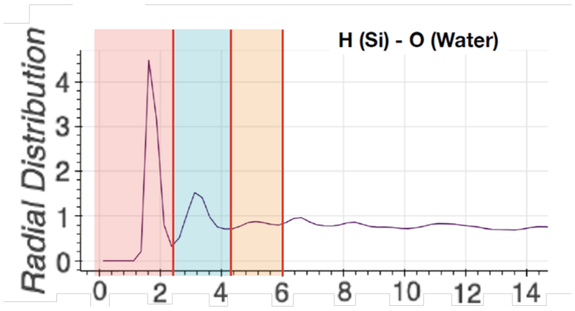

Figure S1: RDF between H_t-O_w of $H_2O/SiO_2/H_2O$ from CMD calculations, the distance on the x axis is in Å. Red lines show the cutoff of different layers used in DFT calculations. Red highlight section is 1 layer of water, blue is 2 layers, and orange is 3 layers.

Strain

The properties of WS_2 in its hexagonal primitive cell were first investigated to obtain a reference for the pristine, unstrained system in vacuum. The band structure for this can be seen in Figure S2a. As expected, a direct band gap of 1.93 eV is calculated. Although this band gap is below the calculated values, this is due to the un-optimized HF %, which is required to allow all materials to be described adequately, as discussed in the methodology.

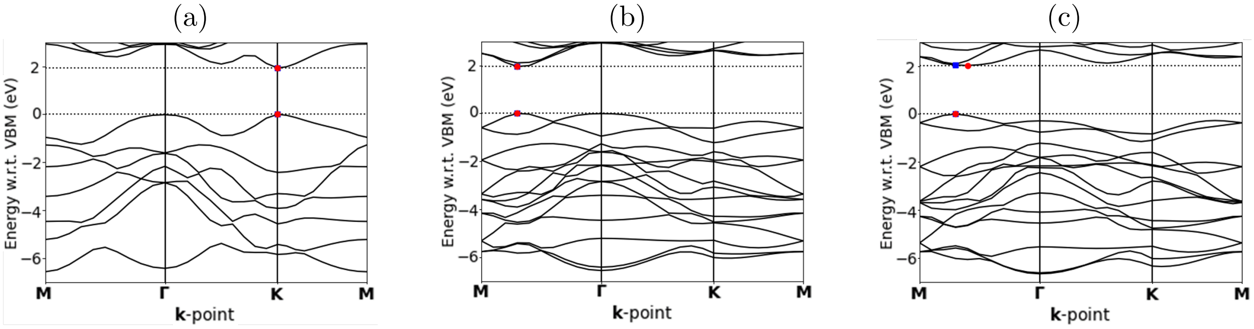

Figure S2: WS_2 band structure under different unit cells and strains. **a)** pristine WS_2 using a primitive hexagonal unit cell; **b)** pristine WS_2 with an orthorhombic unit cell; **c)** strained WS_2 with an orthorhombic unit cell. The red circle indicates the lowest energy difference between the valence and conduction bands. If the lowest energy is an indirect band gap, the direct band gap is shown by a blue square.

WS_2 in the orthorhombic cell was then investigated to understand how the change of the unit cell and, consequently, the Brillouin zone affects the band gap. The band gap of the orthorhombic unit cell is calculated to be 1.95 eV. As expected, the band gap is also direct; this is seen in Figure S2b. The band gap is 0.02 eV higher in the orthorhombic cell than in the hexagonal. This is a minor difference, indicating that the band-gap values will only have a minor error when the cell is changed to an orthorhombic cell.

Finally, the orthorhombic cell was then investigated under strain. To fit the underlying SiO_2 substrate 2.62 % strain (compression) was introduced in the **a** direction and -5.69 % strain (tension) in the **b** direction.

In Figure S2c, the indirect band gap is 2.02 eV, and the direct band gap is 2.04 eV. These two points are close together, both in value and in k-space, so there is a minimal difference between the indirect and direct band gaps. The band gap has a small increase of 0.07 eV compared to the orthorhombic cell and by 0.09 eV compared to the hexagonal cell. Therefore, the introduction of artificial strain into the WS_2 makes the interface structure acceptable.

Average PDoS, with respect to vacuum

For each snapshot PDoS, all eigenvalues were referenced to their vacuum level:

$$\epsilon_f = \epsilon_i - E_{vac} \tag{S1}$$

Where ϵ_f is the final eigenvalue, ϵ_i is the original eigen value, and E_{vac} is the vacuum energy level. The vacuum energy level is set to 0 eV in Figure S3. The snapshots were then averaged and a Gaussian smear of 0.1 was applied.

The hydrogen and oxygen contributions were separated on the basis of the material to which they belonged. H_w and O_w are hydrogen and oxygen from water, H_t and O_t are the hydrogen and oxygen from the top silanol groups that interface with water, H_b and O_b are from the bottom silanol groups that interface with vacuum, and finally, O is from SiO_2.

The band gap of WS_2 can be seen clearly in Figure S3, with the top of the VBM at ≈ 6 eV in all water layers.

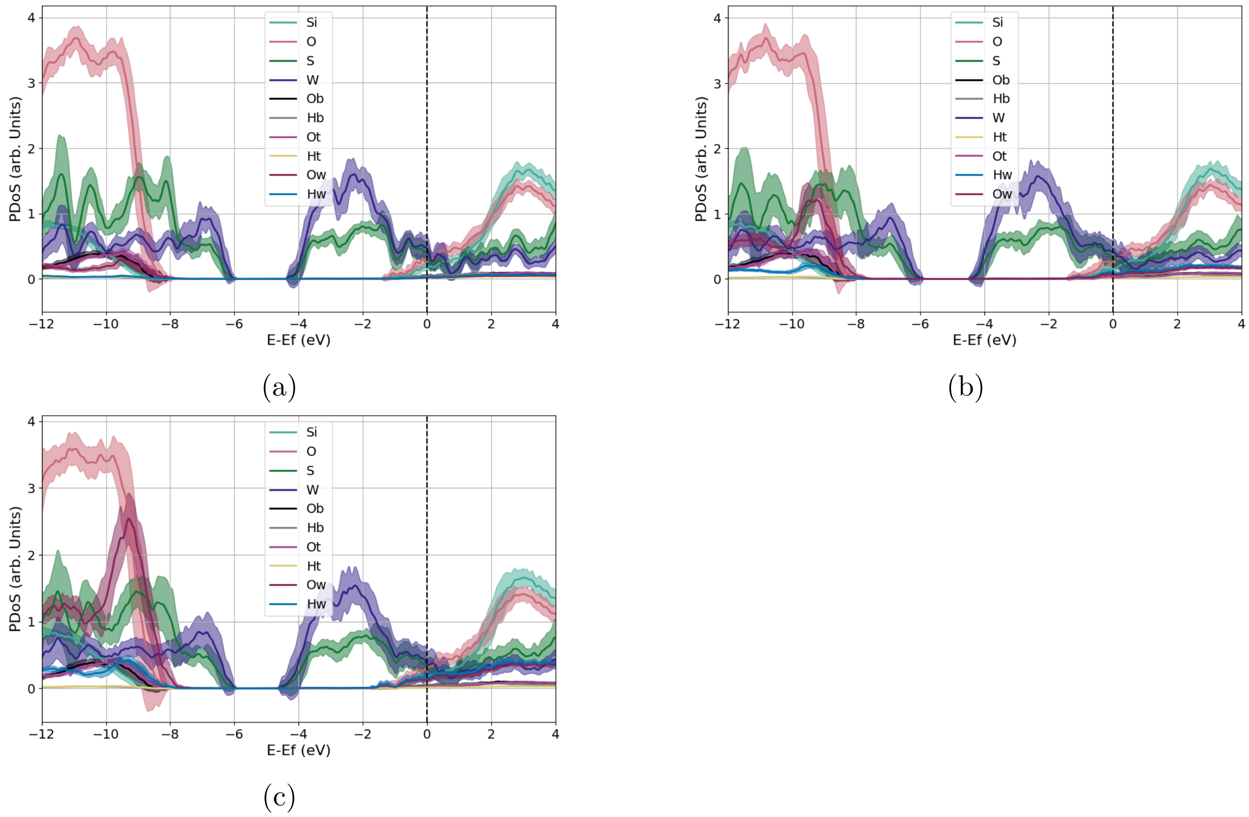

Figure S3: The average PDoS of all snapshots from AIMD calculations. The 0 point marked with a dashed vertical line shows the vacuum energy level. From the systems: **a**) 1 layer, **b**) 2 layers **c**) 3 layers

LDoS

Relative atomic positions

The relative position of the interface $SiO_2/H_2O/WS_2$ atoms compared to the LDoS are shown in Figure S4. The shared axis highlight the averaged interface position compared to the LDoS, with the 0.1 threshold highlighting the spatial dependence of CBM offset and VBM offset. The rest of the SiO_2 band alignment can be seen compared to the 20 Å cutoff as shown in the main text.

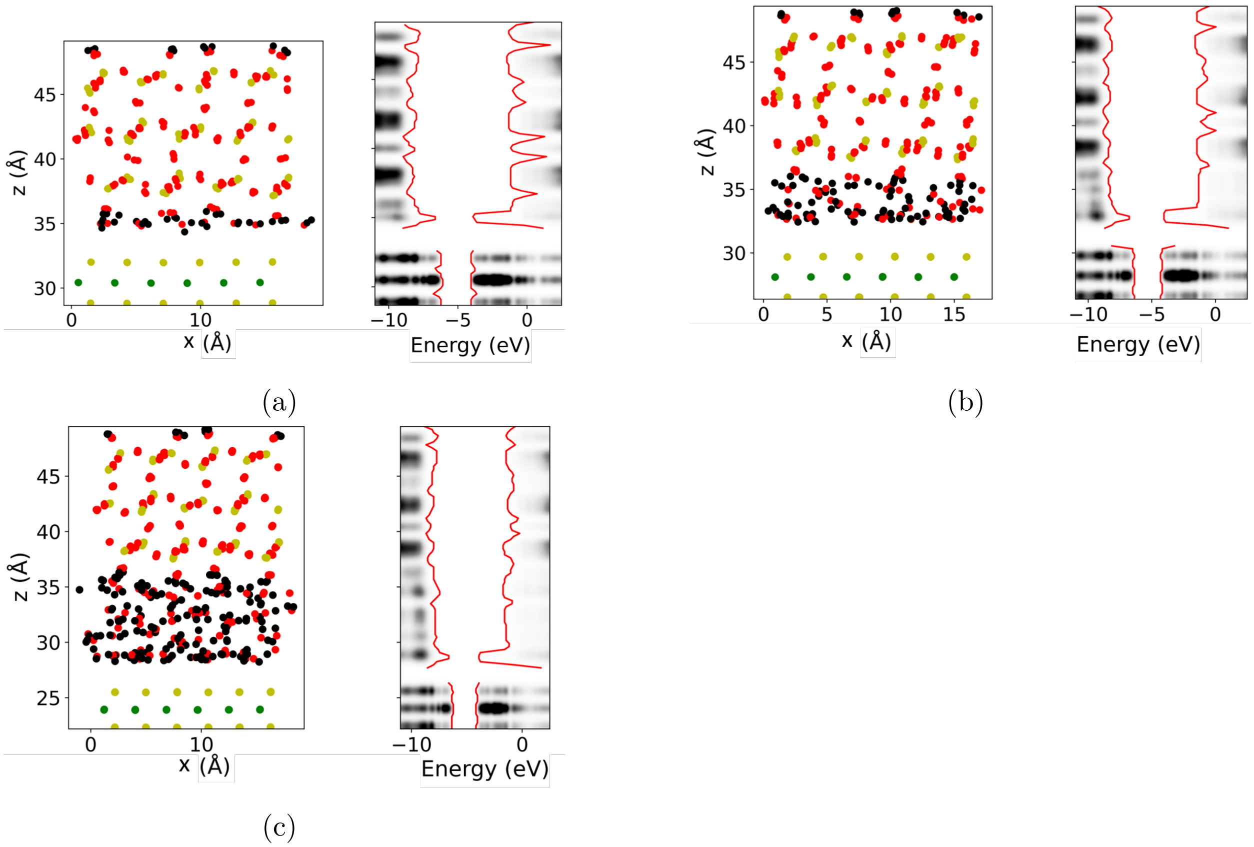

Figure S4: Comparison of average position of the $SiO_2/H_2O/WS_2$ to the corresponding LDoS with a threshold value of 0.1. The systems shown are **a**) 1 water layer, **b**) 2 water layers, **c**) 3 water layers.

Water LDoS

The LDoS from water geometries of each snapshot from the $WS_2/H_2O/SiO_2$ interface were calculated using the same threshold values of 0.1 and 0.5. For all water layers with a 0.1 threshold, the VBM and CBM are consistent across the water so no spatial dependence seen in the LDoS. However, increasing the threshold to 0.5, showed more spatial variation, particularly close to where the SiO_2 interface was previously. We suggest that this is due to the increased density of water molecules close to the SiO_2 and the consequent broken hydrogen bonds in water only snapshots. This change in the VBM and CBM is not seen close to the WS_2 interface as there is less interaction.

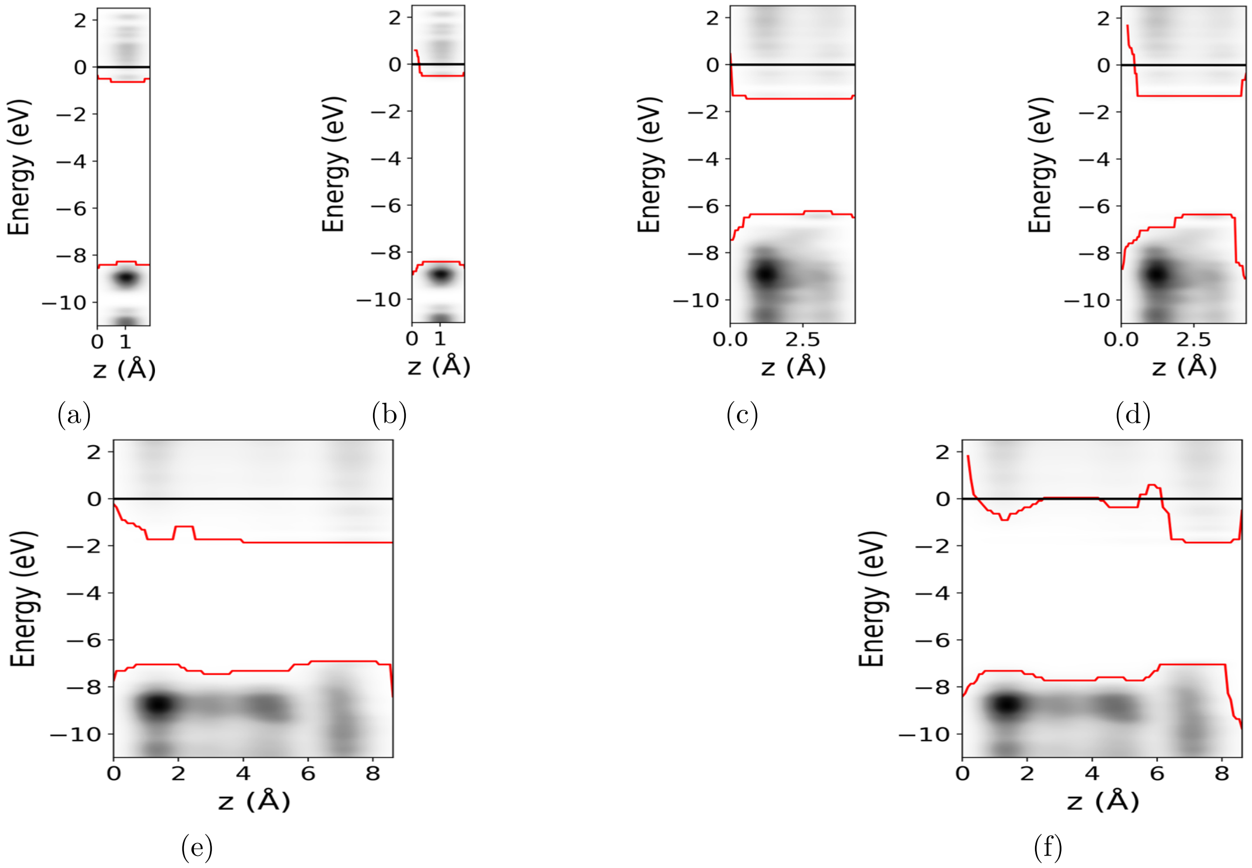

Figure S5: LDoS of water layers: **a,b** 1 layer, **c,d** 2 layers, and **e,f** 3 layers, from averaging AIMD snapshots with a threshold of **a,c,e)** 0.1, and **b,d,f)** 0.5.

Further IPR analysis

The analysis in the main text shows the biggest peak at one snapshot corresponding to the localisation of that wavefunction between water and WS_2. However, there are other localisaed peaks, as seen by peak b in Figure S6a. This peak corresponds to a state localised on the bottom silanol groups of the silica slab (Figure S6b). This highlights the slab nature of the SiO_2 used in this work as although the slab is passivated by the silanol groups, there are still localised states forming due to the termination of the bottom surface.

We also highlight further states that can form at different snapshots, with peak e in Figure S6d showing greater localisation than in f, despite e (Figure S6e) showing a state between SiO_2 and water instead of between WS_2 and water (Figure S6f).

Comparing the position of WS_2 and H_2O state peaks c (-1.98 eV) and f (1.51 eV), we

see that the energy has shifted by 0.47 eV. Therefore, although these water in gap states are present in different snapshots, the energy fluctuates.

The number and variety of localised states at this interface could impact the conduction of WS_2 via the WS_2-water states, but also via additional states formed between the SiO_2 surface and water which may lead to charge moving away from the WS_2.

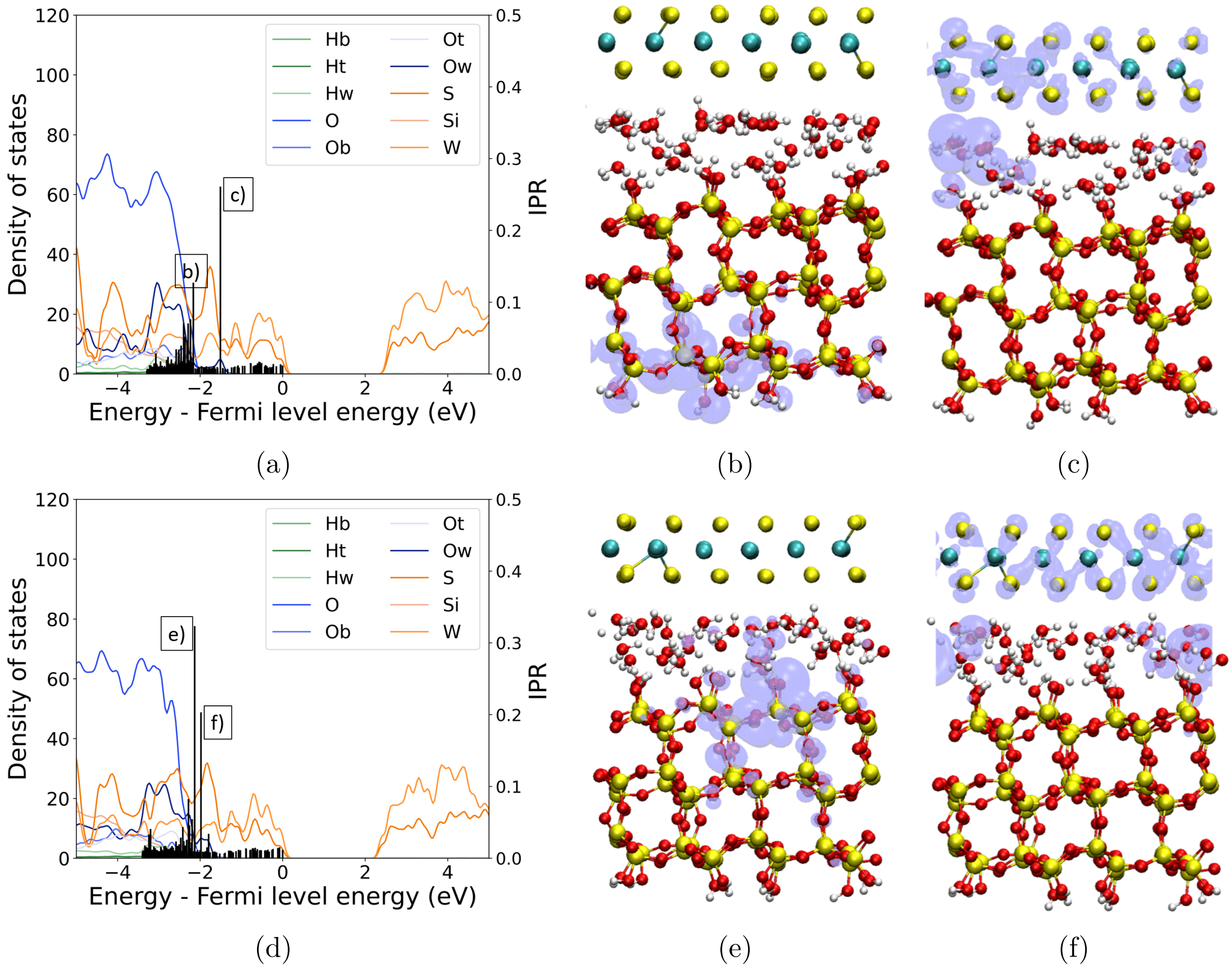

Figure S6: IPR Analysis of two snapshots in the two layer system at **a-c)** 5.5 ps (seen in main text), and **d-f)** 8ps. **a**) and **d**) show the IPR spectrum imposed on the PDoS of the snapshots, with the two largest IPR peaks labelled according to the relevant wfn, i.e. **b**) corresponds to label b in **a**). The Molecular Orbitals shown in **b,c,e,f**) are with isosurface: 0.01.

Charge Transfer

Charge transfer and electrostatic interaction at the $H_2O/WS_2/SiO_2$ interface were calculated using the Bader charge, which was calculated using the code developed by the Henkelmman group.[1]

Bader charges were calculated for each snapshot of the $SiO_2/H_2O/WS_2$ interface to determine the charge transfer between WS_2 and H_2O. The Bader charges of WS_2 and H_2O/SiO_2 were computed based on the geometry of each snapshot from the $SiO_2/H_2O/WS_2$ interface. The average change in charge, resulting from the formation of the interface, was then determined for each atom of interest. In this work, we focused on the interfacial atoms, specifically W and S from WS_2, H and O from H_2O (denoted as H_w and O_w, respectively), and the silanol groups interacting with water on the silica surface, labeled as O_t and H_t. This gave the general formula for change in charge for each atom as:

$$\Delta q = \frac{1}{N_{SS}} \sum_{n=1}^{N_{SS}} q_{(XY)_n} - q_{(X)_n} - q_{(Y_n)}. \tag{S2}$$

Here, q is the charge of the atom, n is the snapshot number, and N_{SS} is the number of snapshots of the AIMD trajectory. XY is the charge on the atom in the entire interface, and X and Y are the Bader charges from the atom in the individual materials at the interface.

The average charge transfer at the $SiO_2/H_2O/WS_2$ interface also highlights the relatively small impact of water on WS_2 on a long time scale. As seen in Figure S7, the charge transfer increases with increasing number of water layers, with the charge transfer spatially dependent in both water and WS_2. The largest magnitude of charge transfer is seen closest to the WS_2 in all models. This aligns with previous band alignment diagrams, showing the spatial dependence of the water valence band aligned with WS_2 close to the interface. The average charge transfer shows that the order of magnitude of the change is small, indicating that this is only a small electrostatic interaction. This is in contrast to the hypothesis suggested by Serron *et al.*[2] that changes in conductivity at the interface is due to charge transfer.

However, in this system, we only consider pristine WS_2 and SiO_2, so any defects that might contribute to charge transfer are not present. Additionally, the systems investigated are not charged or have an applied bias, which may change the magnitude of charge transfer at the interface.

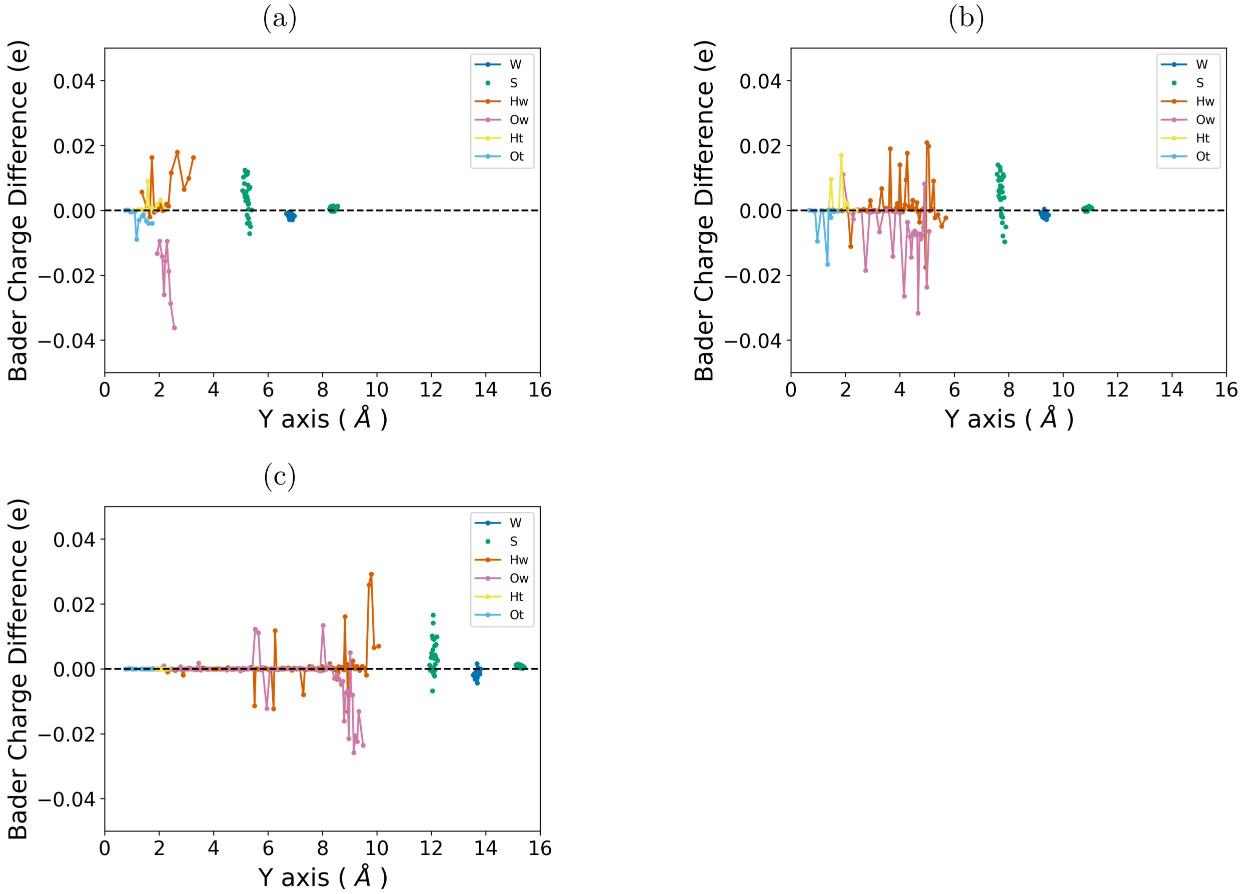

Figure S7: Average Bader charge difference between water and WS_2 at the $SiO_2/H_2O/WS_2$ interface, 0 corresponds to the top of the SiO_2 surface. The Positions of the atoms are averaged over snapshots of the AIMD run. **a)** 1 layer of water, **b)** 2 layers of water, **c)** 3 layers of water. H_w and O_w is the water hydrogen and oxygen respectively, and H_t and O_t are the silanol groups hydrogen and oxygen.

References

(1) Tang, W.; Sanville, E.; Henkelman, G. A grid-based Bader analysis algorithm without lattice bias. *Journal of Physics: Condensed Matter* **2009**, *21*, 084204.

(2) Serron, J.; Minj, A.; Spampinato, V.; Franquet, A.; Rybalchenko, Y.; Boulon, M.-E.; Brems, S.; Silva, H. M.; Shi, Y.; Groven, B.; et al. Conductivity Enhancement in Transition Metal Dichalcogenides: A Complex Water Intercalation and Desorption Mechanism. *ACS Applied Materials & Interfaces* **2023**, *15*, 26175–26189, PMID: 37194926.
